# The timing of antenatal care initiation and the content of care in Sindh, Pakistan

**DOI:** 10.1186/s12884-016-0979-8

**Published:** 2016-07-27

**Authors:** Sohail Agha, Hannah Tappis

**Affiliations:** 1 The Bill and Melinda Gates Foundation, P.O. Box 23350, WA 98102 Seattle, Washington USA; 2 Jhpiego – an affiliate of Johns Hopkins University, Baltimore, Maryland USA

**Keywords:** Maternal health services, Pregnancy, Pregnant women, Prenatal care, Antenatal care, Utilization, Pakistan

## Abstract

**Background:**

Policymakers and program planners consider antenatal care (ANC) coverage to be a primary measure of improvements in maternal health. Yet, evidence from multiple countries indicates that ANC coverage has no necessary relationship with the content of services provided. This study examines the relationship between the timing of the first ANC check-up, a potential predictor of the content of services, and the provision of WHO recommended services to women during their pregnancy.

**Methods:**

The study uses data from a representative household survey of Sindh with a sample comprising of 4,000 women aged 15–49 who had had a live birth in the two years before the survey. The survey obtained information about the elements of care provided during pregnancy, the timing of the first ANC check-up, the number of ANC visits made during the last pregnancy and women’s socio-economic and demographic characteristics. Bivariate analysis was conducted to examine the relationship between the proportion of women who receive six WHO recommended services and the timing of their first ANC check-up. Multivariate analysis was conducted to identify predictors of the number of elements of care provided.

**Results:**

While most women in Sindh (87 %) receive an ANC check-up, its timing varies by parity, education and household wealth. The median time to the first ANC check-up was 3 months for women in the richest and 7 months for women in the poorest wealth quintiles. In multivariate analysis, wealth, education, parity and age at marriage were significant predictors of the number of elements of care provided. Women who received an early ANC check-up were much more likely to receive WHO recommended services than other women, independent of a range of socio-economic and demographic variables and independent of the number of ANC visits made during pregnancy.

**Conclusions:**

In Sindh, the timing of the first ANC check-up has an independent effect on the content of care provided to pregnant women. While it is extremely important that providers are adequately trained and motivated to provide the WHO recommended standards of care, these findings suggest that motivating women to make an early first ANC check-up may be another mechanism through which the quality of care provided may be improved. Such a focus is most likely to benefit the poorest, least educated and highest parity women. Based on these findings, we recommend that routine data collected at health facilities in Pakistan should include the month of pregnancy at the time of the first ANC check-up.

## Background

Antenatal care (ANC) is recognized as a key health service for maternal and child survival, as it presents opportunities to identify conditions that increase risks of adverse pregnancy outcomes, provides a broad range of preventive and curative health services, and builds provider-client relationships [1–4]. Research has shown that women who receive ANC are more likely to have a skilled birth attendant present during delivery - the intervention with the greatest impact on maternal and perinatal mortality [5]. Yet, while the number of women in low and middle income countries attending at least one ANC appointment increased from 63 % in 1990 to 83 % in 2012 (and those attending four or more times rose from 37 % to 52 % over the same period), maternal and neonatal mortality remained high in several countries with satisfactory levels of ANC coverage [6, 7].

The gap between the apparent effectiveness of ANC observed in research studies and changes in mortality observed at the national level has prompted stakeholders to call for a greater focus on the content and quality of care than on levels of coverage [8, 9]. Although national protocols may vary, WHO recommends that women receive a core set of services which include blood pressure measurement, tetanus toxoid vaccination, urine testing, iron folate supplementation, body weight measurement and counselling about danger signs. The WHO also recommends that pregnant women with uncomplicated pregnancies should receive four ANC visits during pregnancy, with the first visit occurring before 14 weeks gestation [10].

In Pakistan, there has been a substantial increase in antenatal care coverage over the last two decades, with the percent of women making at least one ANC visit during pregnancy increasing from 26 % in 1990–91 to 78 % in 2012–13. However, the 2006–07 Demographic Health Survey (DHS) showed that among women who received ANC, only 17 % received six elements of care that should be provided during an ANC visit. By 2012–13, only 28 % of women who had had an ANC check-up received these six elements of care [11]. These findings indicate that monitoring service provision in terms of ANC coverage may mask differentials in the quality of care provided to pregnant women, as quality of care may remain poor while there are large increases in coverage. These findings from Pakistan are consistent with analyses from other low and middle income countries which suggest that there are substantial gaps between ANC coverage levels and the receipt of WHO recommended content of care [9, 12].

A recent analysis of data from the 2006–07 Pakistan DHS found that women’s reasons for the first ANC visit differed by household poverty status. Approximately 71 % women in the poorest quintile made their first ANC visit because they experienced a pregnancy-related problem, while the majority of non-poor women made their first ANC visit for routine preventive care [13]. That the most vulnerable women first seek ANC because of a perceived problem indicates that, for the poorest, least educated women, prevention may not be the motivation for their first ANC visit.

Since the main indicator of antenatal care collected by the Pakistan government’s District Health Information System is how many women make an ANC visit, the lack of precision with which this indicator measures preventive care provided to the most vulnerable women is a matter of importance. If the indicator measuring ANC visits is misleading in terms of preventive care provision, there is a need to consider other potential indicators that may reflect the content of care provided with greater accuracy.

One possible indicator that may be a more precise reflection of the care that women receive is the timing of their first ANC visit. Studies in other low and middle income settings have considered the latter to be an important indicator of ANC quality, as early initiation of ANC allows more time for additional visits and potentially increases the likelihood that a woman will receive the full range of services recommended during pregnancy [14–17]. However, most of these studies have focused on factors associated with timely initiation of ANC or on the relationship between timing of the first ANC visit and specific health outcomes [18–22]. We are not aware of studies that have examined the relationship between the timing and content of care received during ANC. Moreover, while the data on the timing of the first ANC visit is routinely collected in household surveys, there has been relatively little examination of the potential of this indicator to serve as a proxy for the content of care received during pregnancy.

This study looks at the timing of antenatal care initiation in Sindh province, Pakistan, with a view to examining whether the timing of the first ANC visit is associated with the number of WHO recommended preventive services received during pregnancy. An early ANC check-up is likely to be important because women who receive ANC earlier in their pregnancy are potentially more likely to receive the full range of WHO recommended services. To the best of our knowledge, we are not aware of any study conducted in Pakistan that has examined the timing of the first ANC visit and its implications for the content of care.

## Methods

Data for this study were collected as part of a survey conducted to provide data on key maternal, newborn, child and reproductive health indicators in Sindh province in 2013.

### Study setting

Approximately 44 million people live in Sindh province, which includes the five districts of Karachi as well as 22 predominantly rural districts with limited health infrastructure. The 2012–13 Pakistan Demographic and Health Survey found that the neonatal mortality rate in Sindh was 54 deaths per 1,000 live births, the infant mortality rate was 74 per 1,000 live births, and the under-five mortality rate was 93 deaths per 1,000 live births. With a total fertility rate (TFR) of 3.9, Sindh had a TFR higher than the national average of 3.8. In Pakistan, maternal and child health services are provided by a mixed health care delivery system dominated by the private sector, complemented in rural areas by a network of public sector community health workers, including Lady Health Workers and community midwives [11].

### Sampling

The study covered all 27 districts of Sindh. It used a multi-stage stratified sampling design to select 4,000 women who had had a live birth in the two years before the survey [23]. The survey oversampled rural districts of Sindh. Probability proportionate to size sampling was used to select the required number of villages (rural) and city sections (urban) in each selected district. Random sampling was used to identify households within each primary sampling unit (PSU). A total of 494 PSUs were selected for the survey, with 8 interviews being conducted per PSU. Detailed information about the survey methodology is available online [11]. The study provides a representative sample of Sindh.

### Data collection

Data were collected in June and July 2013 through face-to-face interviews with female participants at their homes. A structured questionnaire based on the 2012–13 Demographic and Health Survey instrument for Pakistan developed by Macro International, Inc. was used for the survey [11]. Women were asked to provide basic information such as age, parity and educational status, along with information about the most recent pregnancy and health care received before, during and after delivery. Studies have shown that mothers’ recall of pregnancy-related events is valid and reliable over long periods of time [24]. The questionnaire was translated into Urdu and Sindhi, and data collected by female staff of a survey research firm that has been conducting national-level household surveys in Pakistan for more than 10 years. Verbal consent was obtained from all participants prior to conducting the interview. This study was approved by the Johns Hopkins Bloomberg School of Public Health Institutional Review Board and the National Bioethics Committee of Pakistan.

### Data analysis

#### Outcome variable

The outcome variable of interest for this study is the content of care provided to a woman during ANC visits. Women were asked about the services they were provided during ANC check-ups for their most recent pregnancy. These services included blood pressure measurement, blood sample testing, urine sample testing, body weight measurement, whether they were given iron tablets and whether they received tetanus immunizations. An outcome variable, comprising of a simple count of the number of elements of care received, was created. The variable had a minimum value of zero and a maximum value of six.

#### Independent variable

The key independent variable of interest for this study was the timing of the first ANC check-up. Women were asked how many months pregnant they were when they first received antenatal care for their most recent pregnancy which occurred during the last two years.

#### Control variables

Other independent variables were conceptualized as control variables. Several studies have found important effects of socio-demographic factors on maternal health services utilization. Income and wealth have been found to be powerful determinants of the utilization of maternal health services [25]. Maternal education is thought to influence the utilization of maternal health services through multiple mechanisms: education may increase awareness of the benefits of preventive health; education may also increase a woman’s confidence in dealing with health care providers [26]. Older ages at marriage, a reflection of greater autonomy of women, may be associated with higher use of maternal health services [27]. Although studies have rarely examined the determinants of early initiation of ANC in developing countries, it is likely that socio-demographic factors play some role in early initiation of ANC. Hence, a range of socio-demographic variables are included as control variables.

A binary variable was created measuring urban or rural residence. Categorical variables were created for woman’s age (15–24, 25–34, 35–49), woman’s age at marriage (12–15, 16–20, 21 or older), the number of children (one, two, three, four, five or more), the highest level of school attended (no formal education, primary or middle school, secondary or higher education). Principal component analysis was used to create a variable measuring household wealth. The data used to create this variable were based on the following assets and amenities: ownership of mobile phone, motorcycle, television, refrigerator, cupboard/cabinet, washing machine, bed, clock, sofa, sewing machine, livestock, construction material use for the floor, construction material use for the floor roof, construction material use for the wall, main fuel used by the household, whether the household has a water pump and whether the household has a toilet. This is approximately similar approach to the one used widely by the Demographic Health Surveys [28].

#### Statistical analysis

Data analysis was conducted using Stata 12.1 (StataCorp, College Station, TX.). Weights were attached to the data to adjust for oversampling of rural areas. Bivariate analysis included constructing a Kaplan-Meier survival curve to estimate the time to first ANC check-up. Bivariate analysis examined the relationship between the timing of the first ANC check-up and the content of care provided to women. Bivariate analysis also examined the relationships between socio-demographic variables and whether the first ANC check-up occurred within 3 months of pregnancy.

Ordinary least squares (linear) regression was used to determine whether the timing of the first ANC check-up was associated with a woman receiving higher quality of care during her pregnancy, controlling for socio-demographic factors and the number of ANC visits made during the pregnancy. The SVY suite of commands in STATA was used to conduct weighted analysis and control for clustering of observations at the PSU level. We tested for endogeneity, to see if unmeasured factors (such as a woman’s motivation) may be responsible both for earlier initiation of ANC and for seeking better quality of services. If unmeasured factors are responsible for both the seeking of better quality of services and for earlier initiation of ANC, biased estimates may be produced [29]. However, our analysis found no evidence of endogeneity (not shown).

## Results

Table 1 (Column 1) presents characteristics of women participating in the study. Consistent with Sindh having the highest rate of urbanization in the country, approximately half (49 %) of women in the sample lived in urban areas. Nearly one-third of women (32 %) were aged 15–24. The mean age of respondents in the sample was 27 years (not shown). Nearly one-fourth (23 %) of women had five or more children, with three living children being the average (not shown). More than half (57) of women in the sample had no formal education, 20 had primary or middle level schooling, and 24 % had secondary or higher level schooling. The vast majority of women in the sample (87 %) had received antenatal care during their last pregnancy. As mentioned earlier, the sample comprises of women who had a live birth during the two years prior to the survey.

**Table 1:** Characteristics and percentage of study population who received an ANC check-up during their last pregnancy

	Sample characteristics (*n* = 4,000)	% of women who received an ANC check-up within 3 months of becoming pregnant	
	%	%	Number of women
Residence			
Urban	48.8	59.6^***^	1,951
Rural	51.2	36.2	2,049
Age			
15–24	32.2	51.2^***^	1,288
25–34	55.2	47.8	2,207
35–49	12.6	37.8	505
Age at marriage			
12–15	12.6	33.7^***^	505
16–20	62.6	44.7	2503
21 or older	24.8	62.2	992
Number of living children			
1	24.5	59.6^***^	981
2	23.9	50.7	956
3	17.0	48.1	680
4	11.8	42.9	471
5 or more	22.8	33.4	912
Education			
None	56.6	34.9^***^	2,265
Primary/Middle	19.5	52.7	780
Secondary or higher	23.9	73.6	955
Wealth			
First/poorest	20.0	26.2^***^	801
Second	20.0	33.0	800
Middle	20.0	48.8	801
Fourth	19.9	56.0	798
Fifth/richest	20.0	74.0	800
Received ANC during last pregnancy			
No	13.4	-	-
Yes	86.6	-	-
Total	100.0	47.6	4,000

^***^ *p* < 0.001

### Timing of first ANC check-up

Table 1 (Column 2) shows the percentage of women who received the first antenatal care check-up with 3 months of becoming pregnant. Nearly half of women in Sindh (48 %) received ANC within 3 months of pregnancy. In bivariate analysis, there were substantial differences in the timing of the first ANC check-up by residence and socio-demographic variables. Nearly 60 % of urban women received a check-up within 3 months compared to 36 % of rural women. A higher proportion of women 15–24 received ANC within the first 3 months compared to women 35–49 (51 % vs 38 %). Women married at older ages were more likely to receive ANC within 3 months than women married at younger ages: 62 % of women married at age 21 or older received a check-up within 3 months of pregnancy, compared to 34 % of women married before age 16.

ANC coverage declined with parity: 60 % of women at parity one received ANC within 3 months, compared to 48 of women at parity three and 33 % of women at parity five. The percentage of women who received ANC within 3 months of pregnancy increased dramatically with education and household wealth. About 35 % of women with no education received a check-up within 3 months compared to 74 % of women with secondary or higher education. About 26 % of women in the lowest quintile received ANC within 3 months compared to 49 of women in the middle quintile and 74 % of women in the richest quintile.

**Fig. 1:**
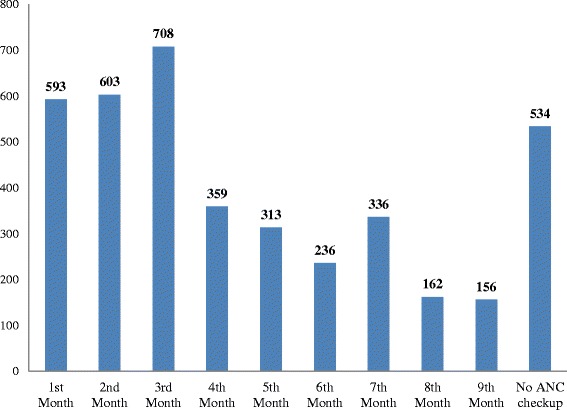
Number of women in Sindh receiving their first ANC check-up by month after becoming pregnant

Figure 1 shows the number of women who received an ANC visit by month after becoming pregnant. Of the 4,000 women in the sample, 48 % (1,904 women) received a check-up by the 3rd month after becoming pregnant. About 22 % (890 women) received an ANC check-up after being pregnant for 6 months or longer. Only 13 % (534 women) did not receive an ANC check-up during their pregnancy.

**Fig. 2:**
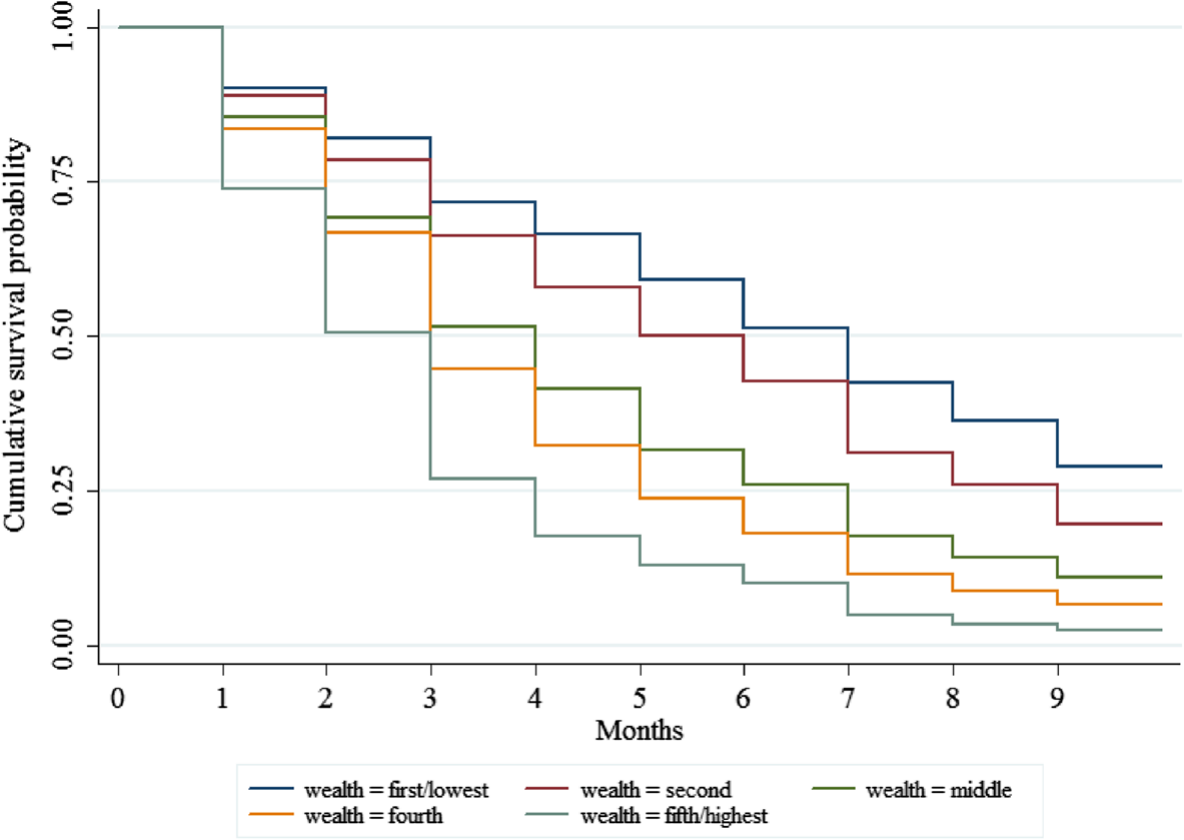
Kaplan-Meier cumulative survival function for time to first ANC check-up by wealth status in Sindh

Figure 2 presents a Kaplan-Meier cumulative survival probability function which shows the fraction of women who received their first ANC check-up by month of pregnancy, by wealth quintiles. We looked at the crossing over of the five survival curves with the probability of half the women receiving an ANC check-up. Among women in the richest/fifth quintile, 50 % received their first ANC check-up within 3 months of becoming pregnant. By comparison, 50 of women in the second quintile received their first ANC check-up within 6 months of becoming pregnant and 50 % of women in the poorest/first quintile received their first ANC check-up within 7 months of becoming pregnant.

### Timing of first ANC check-up and receipt of WHO recommended services

Table 2 presents the percentage of women receiving selected ANC services by month in which the first ANC check-up was received. Women were asked whether they received any of the following services during their pregnancy: blood pressure measurement, blood sample testing, urine sample testing, body weight measurement, iron supplementation, and tetanus immunizations. Blood pressure measurement was the most common service provided (81), followed by urine testing (73), blood sample testing (72), iron supplementation (65), tetanus immunization (61), and body weight measurement (56 %).

**Table 2:** Percentage of women who received selected services by month (after pregnancy) of first ANC check-up

	Blood pressure measured	Blood test taken	Urine test taken	Took iron tablets	2+ tetanus shots	Weight measured	
	%	%	%	%	%	%	Number of women
1st Month	91.9	85.2	88.5	73.4	69.2	64.6	593
2nd Month	87.9	81.5	84.4	70.8	66.1	66.4	603
3rd Month	87.9	81.2	83.9	71.2	67.4	66.7	708
4th Month	83.3	74.2	75.8	66.6	59.6	56.8	359
5th Month	75.0	63.3	60.9	61.3	56.7	49.2	313
6th Month	75.8	63.1	61.9	51.7	54.7	44.1	236
7th Month	72.0	57.0	56.0	60.7	56.2	45.7	336
8th Month	52.8	38.5	40.1	48.8	42.9	22.8	162
9th Month	42.3	30.8	25.6	32.1	39.7	19.2	156
Total	80.9	71.7	73.0	65.0	61.4	55.9	3,466

For every recommended ANC service measured in the survey, there was a steady decline in the percentage of women receiving the service by the month in which they received their first ANC check-up. The percentage of women whose blood pressure was measured during their pregnancy declined from 92 % if they received their first ANC check-up within 1 month of becoming pregnant, to 75 % if they received their first ANC check-up within 5 months of becoming pregnant to 42 % of women who received their first check-up within 9 months of becoming pregnant. In the case of the urine test, the percentage of women declined from 89 % if they received a check-up in their 1st month, to 61 % if they received a check-up in their 5th month to 26 % if they received a check-up in their 9th month. In the case of tetanus toxoid injections, the percentage declined from 69 % to 57 % to 40 % depending upon whether they received their first ANC check-up in the first, fifth or 9th month after becoming pregnant, respectively.

Table 3 presents coefficients from a multiple regression model predicting the number of elements of care received by a woman during her last pregnancy. The number of ANC check-ups made by a woman during her pregnancy was the strongest predictor of the number of elements of ANC received. Household wealth, education, urban residence and age at marriage had significant associations with the number of elements of care received. Even after controlling for these variables, however, the timing of the first ANC check-up was a significant predictor of the content of care received during pregnancy.

**Table 3:** Multiple regression coefficients of the number of elements of ANC quality received, by socio-demographic characteristics, number of ANC visits and timing of first ANC visit

	OLS Coefficients	95 % Confidence Intervals	Number of women
Residence			
Rural	ref		1,951
Urban	0.38^***^	0.19–0.57	2,049
Age at marriage			
12–15	ref		505
16–20	0.19^*^	0.02–0.36	2503
21 or older	0.32^**^	0.12–0.52	992
Number of living children			
1	ref		981
2	−0.05	−0.18–0.07	956
3	0.01	−0.13–0.15	680
4	−0.09	−0.27–0.08	471
5 or more	−0.16^*^	−0.31–0.01	912
Education			
None	ref		2,265
Primary/Middle	0.36^***^	0.19–0.52	780
Secondary or higher	0.61^***^	0.45–0.77	955
Wealth			
First/poorest	ref		801
Second	0.21^***^	0.03–0.40	800
Middle	0.66^***^	0.43–0.88	801
Fourth	0.90^***^	0.65–1.16	798
Fifth/richest	1.08^***^	0.82–1.35	800
Number of ANC visits			
None	ref		533
1–3	2.12^***^	1.95–2.28	1412
4 or more	3.20^***^	2.99–3.40	2054
Timing of first ANC visit			
> 3 months or no visit	ref		2095
Within 3 months	0.36^***^	0.22–0.49	1905
R-squared	59.9 %		4,000

^*^ *p* < 0.05, ^**^ *p* < 0.01, ^***^ *p* < 0.001

## Discussion

Consistent with data from the Demographic and Health Surveys of Pakistan, which have shown tremendous increases in ANC coverage in Pakistan between 1990 and 2012, our study showed that 87 % of women in Sindh had received at least once ANC check-up. Even among women in the poorest wealth quintile, ANC coverage was substantial (70 %). These levels of ANC coverage do not, however, correspond to the alarmingly high level of neonatal mortality or the slow pace of reduction of maternal mortality observed in Pakistan. This may, in part, be due to the fact that ANC coverage by itself does not predict the content of care provided to women during pregnancy [9].

There is an urgent need for an indicator which provides a more accurate picture of the preventive care provided to women during pregnancy. We examined the timing of the first ANC check-up as a possible predictor of the content of care provided to pregnant women. While the importance of this indicator has been overlooked in the literature, data on this indicator is collected across many standard surveys including the Demographic Health Surveys. Our study found that the timing of the first ANC check-up is a powerful predictor of the content of services. Women who received their first ANC check-up within 3 months of becoming pregnant were significantly more likely to receive WHO recommended services during their pregnancy.

ANC coverage masks large differentials by wealth, education and parity in the timing of the first ANC check-up. For example, there is a median time difference of 4 months in the timing of care initiation between the poorest and wealthiest women: the median time for the first ANC check-up is 7 months after pregnancy among the poorest women, compared to 3 months after pregnancy among the wealthiest women. Parity and education have similar strong effects on the timing of the first ANC check-up.

These results have important implications for countries such as Pakistan where ANC is promoted as a key intervention and ensuring high ANC coverage is a priority of the national health program. Yet, in Pakistan, relatively little attention is paid to the content of care provided. The results from this study show that there is considerable variation in the preventive services a woman receives during ANC. If a woman receives ANC relatively early in her pregnancy, she is much more likely to receive the full range of services recommended by WHO compared to a woman who receives ANC after four or 5 month of being pregnant.

The findings of this study suggest that informing women of the need to get an early ANC check-up is likely to increase their chances of getting the WHO recommended content of care.

Programs should motivate the most vulnerable women – women with low education and at high parity - to make an ANC visit as early as possible after becoming pregnant. By communicating the importance of an early first ANC visit, programs are likely to have greater success in ensuring that pregnant women receive the recommended content of care. At present, in Pakistan there is little focus on the content of care provided to women or on the timing of the first ANC visit. A shift in programmatic focus towards an early ANC check-up may increase the strength of the relationships between ANC and reductions in neonatal and maternal mortality – relationships that appear to be extremely weak at present.

Of course, encouraging early and regular attendance is not sufficient to ensure that women receive quality ANC. Reasons for a woman’s delaying initiation of ANC may include her not being informed about when to make the first visit, difficulty in accessing care and concerns about the manner in which the provider delivers care [15, 16, 30, 31]. Efforts to promote and enable early and regular care-seeking must be coupled with efforts to ensure that services are accessible, that the ANC visit is a positive experience for pregnant women and is provided in a client-friendly environment by practitioners with good clinical and interpersonal skills [32, 33].

The findings of this study also have important implications for the collection of routine data at health facilities. The number of ANC visits is one of the key performance indicators for public health facilities in Pakistan and is considered an indicator of the efficiency of health facilities and a proxy for improvements in maternal health outcomes [34, 35]. Our findings suggest that the routine health information system in Sindh should collect data on the timing of the first ANC check-up rather than simply collecting information on whether an ANC check-up occurs. At present the district health information system only collects data on whether a first ANC check-up is conducted and whether a repeat ANC visit occurs [36]. Data on the timing of the first ANC check-up may serve as a useful proxy for the content of care provided.

### Strengths and limitations

This study has the same limitations as other observational studies which are based on cross sectional data. Since data on dependent and independent variables was collected at the same point in time, no causal interpretation can be made of the relationships between variables. The data are based on self-reports from women, which may be subject to recall and/or courtesy biases. However, previous studies have shown that maternal recall of pregnancy-related events has high validity and reliability. Moreover, the short recall period of this study is also likely to minimize any recall bias. Another limitation is that this study does not collect information on the full range of services recommended by WHO for a pregnant woman. It is possible that other WHO recommended preventive services do not follow the same pattern observed for services measured in our study.

Strengths of this study include a shorter recall period compared to surveys such as the Demographic and Health Surveys: in our study, women are asked about antenatal care during their last pregnancy which resulted in a live birth that occurred during the previous two years. A shorter recall period lowers recall bias inherent in reported information. Another strength is the large sample size of this survey (*n* = 4,000) for Sindh province. We are not aware of any other provincially representative maternal, neonatal and child health survey conducted in Sindh in recent years which has a comparable sample size.

## Conclusions

In Sindh, the timing of the first ANC check-up is strongly associated with the content of care provided to pregnant women. Given the cross-sectional nature of the data used for this study, it is not possible to draw firm conclusions regarding the relationship between the timing of the first ANC visit and the number of elements of care received by women during pregnancy. Experimental studies are needed to investigate whether the timing of the ANC visit does indeed influence the content of care received by pregnant women. If experimental studies support our findings, it will increase the range of options available to programs to improve the quality of service provision during pregnancy. At present, the primary option available to programs is to ensure provider adherence to standards of care provision. Yet, currently, there is little evidence of interventions that consistently improve provider adherence to standards of care in developing countries. Studies show that the quality of ANC continues to be poor in many developing countries, including in Pakistan. Our findings suggest that motivating women to make an early first ANC check-up may be a possible demand-side approach that may help in improving the quality of care they receive. Such an approach is most likely to benefit the poorest, least educated and highest parity women.

It is extremely important that data collection systems in Pakistan include data on the timing of the first ANC visit rather than whether an ANC visit occurs. To provide a better measure of performance, routine data collected at health facilities in Pakistan should include the month of pregnancy at the time of the first ANC check-up.

## Abbreviations

ANC, antenatal care; WHO, World Health Organization
